# Prospective, multicenter, controlled study of quality of life, psychological adjustment process and medical outcomes of patients receiving a preemptive kidney transplant compared to a similar population of recipients after a dialysis period of less than three years – The PreKit-QoL study protocol

**DOI:** 10.1186/s12882-016-0225-7

**Published:** 2016-01-19

**Authors:** Véronique Sébille, Jean-Benoit Hardouin, Magali Giral, Angélique Bonnaud-Antignac, Philippe Tessier, Emmanuelle Papuchon, Alexandra Jobert, Elodie Faurel-Paul, Stéphanie Gentile, Elisabeth Cassuto, Emmanuel Morélon, Lionel Rostaing, Denis Glotz, Rebecca Sberro-Soussan, Yohann Foucher, Aurélie Meurette

**Affiliations:** EA 4275 SPHERE, methodS in Patient-centered outcomes and HEalth ResEarch, Nantes University, Nantes, France; Biostatistics Unit, CHU Nantes, Nantes, France; ITUN and Inserm U1064, Nantes University, CHU Nantes, Nantes, France; Délégation à la recherche clinique et à l’innovation, CHU Nantes, Nantes, France; Laboratoire de santé publique, SPMC EA3279, Aix-Marseille université, 13385 Marseille, France; Service de santé publique et information médicale, hôpital de la Conception, 13005 Marseille, France; Service de Néphrologie, Hôpital Pasteur, Nice, France; Néphrologie, Transplantation et Immunologie Clinique, Hôpital Edouard Herriot, Lyon, France; Department of Nephrology, Dialysis and Transplantation, Hôpital de Rangueil, Toulouse, France; Hôpital Saint Louis - Nephrology and Transplantation, Paris, France; Department of Nephrology and Transplantation, Hôpital Necker Assistance Publique-Hôpitaux de Paris, Université Paris Descartes Sorbonne Paris Cité, Paris, France

**Keywords:** Tansplantation, Nephrology, Preemptive, Dialysis, Quality of Life, Response shift, Item Response Theory, Structural Equation Modeling

## Abstract

**Background:**

Treatment of end stage renal disease has an impact on patients’ physical and psychological health, including quality of life (QoL). Nowadays, it is known that reducing the dialysis period has many advantages regarding QoL and medical outcomes. Although preemptive transplantation is the preferred strategy to prevent patients undergoing dialysis, its psychological impact is unknown. Moreover, transplantation can be experienced in a completely different manner among patients who were on dialysis and those who still had a functioning kidney at the time of surgery. Longitudinal data are often collected to allow analyzing the evolution of patients’ QoL over time using questionnaires. Such data are often difficult to interpret due to the patients’ changing standards, values, or conceptualization of what the questionnaire is intended to measure (e.g. QoL). This phenomenon is referred to as response shift and is often linked to the way the patients might adapt or cope with their disease experience. Whether response shift is experienced in a different way among patients who were on dialysis and those who still had a functioning kidney at time of surgery is unknown and will be studied in the PreKit-QoL study (trial registration number: NCT02154815). Understanding the psychological impact of pre-emptive transplantation is an important issue since it can be associated with long-term patient and graft survival.

**Methods/Design:**

Adult patients with a pre-emptive transplantation (n = 130) will be prospectively included along with a control group of patients with a pre-transplant dialysis period < 36 months (n = 260). Only first and single kidney transplantation will be considered. Endpoints include: comparison of change between groups in QoL, anxiety and depressive disorders, perceived stress, taking into account response shift. These criteria will be evaluated every 6 months prior to surgery, at hospital discharge, at three and six months, one and two years after transplantation.

**Discussion:**

The PreKit-QoL study assesses and compares the evolution of QoL and other psychological criteria in preemptive and dialyzed patients taking patients’ adaptation into account through response shift analyses. Our study might help to conceive specific, adapted educational programs and psychological support to prevent a possible premature loss of the kidney as a consequence of non-compliance in patients that may be insufficiently prepared for transplantation.

**Trial registration:**

ClinicalTrials.gov identifier NCT02154815, registered on May 28, 2014

## Background

### End stage renal disease, dialysis and transplantation

Chronic kidney disease can lead to end stage renal disease (ESRD), which requires use of substitution techniques such as hemodialysis, peritoneal dialysis or kidney transplantation. Treatment of ESRD is cumbersome in terms of implementation, impact on the physical and psychological health of patients, quality of life (QoL) and it generates substantial health expenditures. It is well-known that kidney transplantation in eligible ESRD patients is the treatment of choice compared to long-term dialysis [1] since it is associated with longer survival [2]. Furthermore, the cost of transplantation is lower as compared to dialysis. Some studies have also described higher QoL for kidney transplant recipients compared to patients undergoing dialysis [3, 4] as well as more positive perceptions of illness when changing from dialysis to transplantation [5]. Hence, limiting the dialysis period before transplantation has several advantages in terms of medical outcomes, QoL and costs. In respect with the same arguments, one can expect that preemptive transplantation, i.e. the patient is placed on the transplant waiting list and transplanted prior to progression to ESRD, would be the preferred strategy. Nevertheless, the literature does not provide a clear answer regarding the benefit of preemptive transplant and even less is known about its psychological impact and its possible burden for patients: for instance, patients may not be aware of the benefit of transplantation compared to dialysis but in contrast may suffer from the consequences of transplantation. Although it is recommended to enlighten patients of all possible consequences and complications after the transplantation, patients are sometimes anyway startled since for instance they suffer after the surgery, or are uncomfortable with the load of the immunosuppressive therapy. Furthermore, in transplantation centers, it has been observed that patients receiving a preemptive transplant may consider or perceive transplantation as an event that has deteriorated their well-being [6].That may explain the high variability of preemptive transplant practice worldwide [7]. This lack of data regarding the psychological impact of preemptive transplantation constitutes a true issue since these subjective outcomes can be associated with the long-term patient and graft survival [8]. This issue is important regarding the collective impact of early registration on waiting list: preemptive transplantations are associated with an increase in the duration on waiting lists, which remains an important risk factor of graft failure. In fact, no data allows concluding on post-transplant QoL in particular because transplantation can be experienced in a completely different manner among patients who were on dialysis and those who still had a functioning kidney at the time of surgery.

### Psychological impact of dialysis and transplantation

Studies assessing the psychological impact and QoL of Patients with Preemptive Transplantation (PPT) compared with Patients with Dialysis before Transplantation (PDT) are lacking and it cannot be assumed that preemptive transplantations systematically improve patients’ QoL. Indeed, if few disease limitations and symptoms were experienced before transplantation, the aftermath of the graft may seem disproportionate, especially in case of post transplantation complications (infection, acute rejection, etc.). Moreover, these patients in ESRD who are not on dialysis have to simultaneously be psychologically prepared for dialysis and transplantation, without knowing which of the two treatments they will have to face first. Some patients may prepare for dialysis and then be referred for a transplant instead, for which they are not ready. In contrast, other patients might await transplantation and perceive dialysis as a failure. In this setting, a better understanding of how patients anticipate, live and face pre and post-transplantation and its consequences can contribute to enhance and better orientate educational programs to improve patient care.

### Longitudinal data and response shift

The importance of studying longitudinal evolution of perceived QoL of patients with chronic disease and in identifying its possible associated factors is now well admitted [9–11]. Such QoL data are often collected through questionnaires including different items called Patient-Reported Outcomes (PRO). Despite the fact that PRO are nowadays considered simple, valid and reliable measures of QoL, it is however clear that the cognitive processes involved in completing them are quite complex [12] and that longitudinal PRO data might sometimes be very difficult to interpret. Indeed, some studies have evidenced a stable or even a higher level of perceived Qol in patients with severe chronic conditions, despite a poor physical health state, as compared to the general population [13–15]. By contrast, others have shown opposite results for stable patients, regarding their health status across occasions, reporting changes in QoL. This phenomenon, which might be regarded as a paradox, is sometimes referred to as response shift (RS) [16, 17]. In case of RS, there is a change in the meaning of what the PRO (QoL, anxiety, pain, etc.) is intended to measure over time or in the patient’s expectations and life priorities according to the disease evolution. It is often linked to the way the patients might adapt or cope with their disease experience. RS can result from three different processes [16, 17]: 1) changes in the patient’s internal standards of measurements called recalibration (i.e. the level of QoL or anxiety measured by the questionnaire can be interpreted differently before and after transplantation), 2) changes in the patient’s values and in the relative importance given to the questions or dimensions of QoL that are appraised before and after transplantation, called reprioritization, and 3) changes in the patient’s definition of what is being measured over time called reconceptualization. Hence, it might be problematic or even impossible to distinguish, without appropriate methods, true QoL changes from confounding RS effects. Indeed, patients may give different answers to the PRO over time, not only because their health has changed, but also because their perception on what health or QoL means to them has changed. The accurate assessment of the effect of transplantation for instance can then be obfuscated by RS and lead to biased results, poor power to detect effects of interest and therefore, erroneous conclusions [18–21]. Indeed, RS can seriously alter the psychometric properties of PRO such as reliability, validity and ability to detect true changes (responsiveness) [22]. On the other hand, one can also highlight the therapeutic importance that the phenomenon of RS constitutes in itself by allowing a better understanding of how patients adjust to their illness. RS may well also be one of the goals of therapy in helping patients to cope with their illness and to live with it. Therefore, RS can be viewed either as a nuisance (measurement bias) or as an indication of a possible therapeutic benefit coming from some form of psychological adaptation or adjustment. In fact, it is very likely that the post-transplant QoL experienced by patients for whom first replacement therapy was transplantation might be lower than for patients who have undergone dialysis, even for a short period of time [1, 23]. The rigorous comparison of change in the QoL of preemptive kidney transplant patients and patients who were on dialysis requires taking into account the very likely occurrence of the RS phenomenon, not only in terms of detection, but also in terms of statistical adjustment in the analysis of changes in PRO data.

### Aims and objectives

Our hypothesis is that preemptive kidney transplant recipients have a lower quality of life and present more difficulties regarding psychological adaptation after transplantation compared to patients who were under dialysis before surgery for less than 3 years. The designed project aims at providing a better assessment and understanding of the psychological impacts and of patients’ psychological adaptation, taking into consideration the response shift, as well as patient and graft outcomes following preemptive transplantation. This could help optimizing the management of these patients, particularly regarding therapeutic education programs and adapted implementation of psychological support.

## Methods/Design

### Study design

**Fig. 1:**
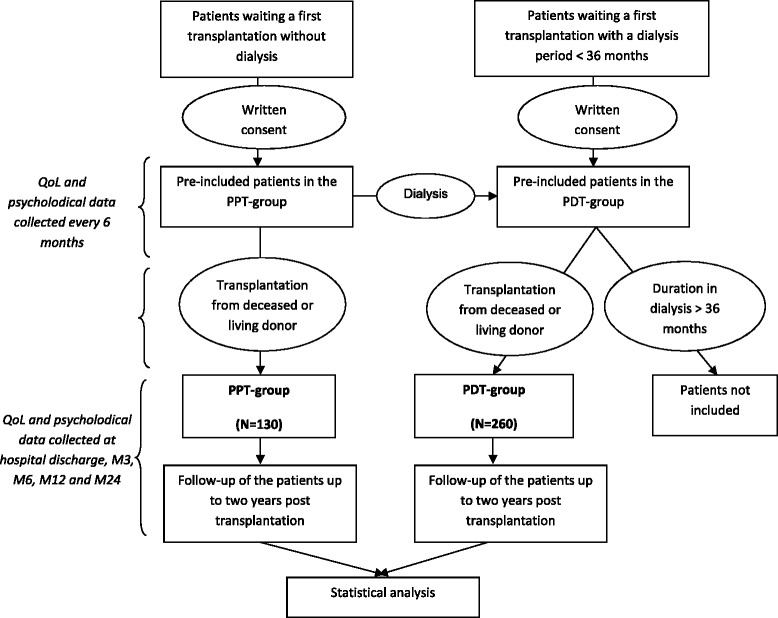
Flowchart of the PreKiT-QoL study. Mx: x months after transplantation, x = 3, 6, 12, and 24

The PreKit-QoL study is a prospective, multicenter, controlled study of quality of life, psychological adjustment process and medical outcomes of patients receiving a preemptive kidney transplant (PPT group) compared to a similar population of recipients after a dialysis period of less than three years (PDT group). The clinical and psychological data as well as the QoL questionnaires will be prospectively collected in the PPT and the PDT groups. Both groups will be matched regarding the recipient gender and the transplantation centers which are the two parameters differentially distributed between the two groups in our prospective cohort DIVAT (www.divat.fr). The type of donors (deceased or living) will be also matched between PDT and PPT. Since it is important to assess QoL before transplantation in order to evaluate response shift related to transplantation, the questionnaires will be collected for all patients waiting a first transplantation without dialysis or with a dialysis period of less than 36 months. Data will be collected at transplantation (sex, age, immunization, medical history, etc.). Other data that change over time, such as QoL, will also be collected every six months before transplantation, at hospital discharge, and 3, 6, 12 and 24 months after transplantation. The flowchart of the study is presented in Fig. 1.

### Study setting and participants

Patients will be mainly recruited from the University Hospital centers participating in the DIVAT network (*Données Informatisées et VAlidées en Transplantation*, 6 centers in France: Nantes, Paris Necker, Paris Saint-Louis, Nice, Lyon). The University Hospitals of Marseille and Toulouse will also participate in the study. The duration of inclusion will be four years. Data collection will take place over six years including the 2-year follow-up of the last included patient. In total, the study will take place over a period of seven years to allow for data analysis. It is a non-interventional research study (routine care), all investigators have agreed to provide patients with clear and precise information about the protocol and will give a copy of the information form to the patients. The patient will be enrolled in the study after reading the information form and signing and dating it. Both documents will be issued in duplicate hard copy format so that the patient and the investigator can each keep a copy. The investigator’s original will be placed in the investigator’s file.

### Inclusion criteria

All patients will be over 18 years old without being under guardianship. All patients with a preemptive transplantation from deceased or living donors will be prospectively included in the PreKit-Qol study. Only first kidney transplantations without other transplanted organ will be considered.

### Primary outcome measure

The primary outcome will be the change in QoL, taking into account the possible occurrence of response shift. This criterion will be measured by a specific questionnaire ReTransQol developed for kidney transplant recipients [24]. It will be evaluated every 6 months prior to surgery, at hospital discharge, and three months, six months, one year and two years after the transplantation.

#### Secondary outcome measures

The following outcomes will be compared between PPT versus PDT:

- The change in generic QoL, subjective well-being, perceived anxiety and depressive disorders as well as perceived stress during follow-up (every 6 months before the surgery, at hospital discharge, 3, 6, 12, and 24 months post-transplantation) taking into account the possible occurrence of response shift. Generic QoL will be assessed with the SF-36 questionnaire. This questionnaire has the disadvantage of not being specific, and therefore less sensitive to specific changes in the health status of the recipient. However, it allows a comparison of the results to a reference population [25] and allows an easier comparison of the patients in different therapeutic frameworks (for example patients before and after transplantation). Subjective well-being, anxiety and depressive disorders, and perceived stress will be assessed using the satisfaction with life scale (SWLS) [26, 27], the Hospital Anxiety and Depression scale (HAD) [28, 29], and the Perceived Stress Scale (PSS) [30], respectively.
- Coping strategies (process of managing stressful circumstances) and post-traumatic growth (positive psychological change experienced as a result of the struggle with highly challenging life circumstances) at 6 and 24 months post-transplantation. Coping strategies and post-traumatic growth will be assessed using with the Brief Cope [31] and the Posttraumatic Growth Inventory (PTGI) [32], respectively.
- The post-transplantation graft and patient outcomes collected at hospital discharge, and 3, 6, 12 and 24-months post-transplantation: i) evolution of the Graft Filtration Rate (GFR) that will be estimated using the 4-variables MDRD formula [33], ii) evolution of the daily proteinuria that will be diagnosed by a simple dipstick test.
- The Kidney Transplant Failure Score at one year post-transplantation [34]. The KTFS is able to predict death-censored graft survival.
- The post-transplantation time to return to work, if appropriate.
- The compliance of patients will be assessed every six months prior to surgery, at hospital discharge, and at 3, 6, 12, and 24 months with the Girerd questionnaire [35].

In addition, other collected clinical data as well as variables related to the social status of the recipient are given in Table 1. The schedule of the study is presented in Table 2.

**Table 1:** Clinical variables directly extracted from the DIVAT data bank

Recipient	Age, gender, weight, height, clinical center, initial disease (glomerulonephritis, diabetes, etc.), cold ischemia time, HLA A-B-DR incompatibilities, number of post transplantation dialyses, pre-transplantation anti HLA immunization whatever the technique used within the six months prior to surgery, cardiovascular history, cancer history, hypertension history, diabetes history, pre transplantation dialysis technique, previous educational program.
Donor	Age, gender, last serum creatinine level, cause of death, hypertension history.
Dates	Birth, first dialysis, registration on waiting list, transplantation, acute rejection episodes, return to work, return in dialysis, last follow-up, death.
The biological follow-up at hospital discharge, three, six, 12 and 24 months	The creatininemia (1) and the daily proteinuria.

(1). The creatininemia will also be collected before the surgery in order to adjust our analysis on the renal function: the psychological impact of the transplantation may depend on the previous grade of renal insufficiency

**Table 2:** Schedule of the PreKiT-QoL study

Activities	Every six months before transplantation	Hospital discharge	M3	M6	M12	M24
Demographic data		X				
Previous medical history		X				
Transplantation data		X				
Donor data		X				
Creatininemia and proteinuria		X	X	X	X	X
ReTransQol questionnaire	X	X	X	X	X	X
SF36 questionnaire	X	X	X	X	X	X
Satisfaction with Life Scale (SWLS) questionnaire	X	X	X	X	X	X
The Perceived Stress Scale (PSS)	X	X	X	X	X	X
The Brief Cope				X		X
The Hospital Anxiety and Depressions Scale (HAD)	X	X	X	X	X	X
The Post-Traumatic Growth Inventory (PTGI)				X		X
Compliance questionnaire	X	X	X	X	X	X
Kidney Transplant Failure Score (KTFS)					X	

X: collected data. Mx: x months after transplantation, x = 3, 6, 12, and 24X: collected data. Mx: x months after transplantation, x = 3, 6, 12, and 24

### Sample size

Computation of a minimum required sample size is not statistically feasible for this study based on the observation of perceived QoL and psychological data taking into account the possible occurrence of RS. Indeed, the concept of RS is complex and novel in this disease and we have no preliminary data or any clear expectation for the results. The sample size will therefore be driven by the inclusion capacities of each center involved in the PreKiT-QoL study. Based on the extraction of the DIVAT data bank of the recipients transplanted between 2010 and 2012, one can expect that at least 130 recipients with preemptive transplantation (case) can be included during the four years inclusion period of the protocol. For each case, two control recipients who have experienced a pre-transplant dialysis period of less than 36 months will be included matched on gender, transplant center, and type of donor. A total of 390 patients will be included. This ratio 1:2 has been chosen to increase the sample size and the statistical power, the main expected limitation being the inclusion of patients receiving preemptive transplantation.

### Statistical analysis

Recipients will be described according to both groups, PPT and PDT. Such descriptive analyses will be performed on the main socio-demographic and clinical variables, either related to the recipient (except for the gender which is a matched between both groups), the donor or the transplantation. Categorical variables will be compared using Mac Nemar Chi2 tests for matched data. Continuous variables will be compared using a Student’s t-tests for paired data. This step will be important to assess the heterogeneity of the two populations and to identify possible confounding factors.

Structural equation models (SEM) and models coming from Item Response Theory (IRT) will be used to analyze changes in QoL and psychological dimensions (subjective well-being, perceived anxiety and depressive disorders, perceived stress) including the detection of response shift (RS) occurrence and its adjustment in the analyses, where appropriate, to estimate true changes. SEM and IRT will be used as complementary methods since SEM is appropriate for detecting RS at the dimension level (e.g. the “Physical Health” dimension of the RetransQoL) and IRT is appropriate for detecting RS at the item level within a dimension (e.g. the item “You are as well as anyone else” in the “Physical Health” dimension of the RetransQoL). SEM models [36] often include variables that are not directly observed (latent variables corresponding to QoL dimensions for instance) and possible links between these and the observed variables (observed scores for each QoL dimension). IRT models belong to the family of generalized linear random effects models [37]. They describe the probability of responding to each response category of a given item as a function of the latent variable one is trying to measure (patient’s “true QoL”) taking into account the psychometric characteristics of the scale through the questionnaire’s items parameters. Identification of RS and “true change” estimation (change in the subjective criterion being studied taking RS into account) will be performed in the following way: SEM analyses will follow the iterative algorithm developed by Oort [38] and IRT analyses will be done using a specific iterative algorithm called the RespOnse Shift ALgorithm in Item response theory (ROSALI) that has been recently developed [39].

Change in coping strategies and in post-traumatic growth will be measured at 6 and 24 months after transplantation and assessed using linear mixed models [40]. The GFR will be measured at hospital discharge, and 3, 6, 12, and 24 months post-transplantation and estimated using the MDRD formula [33]. A linear mixed model will also be used to handle these longitudinal measurements. Three explicative variables will be systematically taken into account: post-transplantation time, preemptive transplantation (yes/no) and the interaction between them. According to the non-linear relationship between the post-transplantation time and the GFR evolution [41], two slopes will be modeled with a breakpoint at 6 months. For all linear mixed models, random terms will be included on the intercept, on the slopes or both. Univariate (p < 0.2) and multivariate (p < 0.05) analyses will be performed in order to take into account possible confounding factors. The daily proteinuria will be measured at hospital discharge, and 3, 6, 12 and 24 months post-transplantation. This random variable is not Gaussian. A Gamma distribution is more appropriate but since a generalized linear models with such an inverse function as canonical link function will be difficult to interpret, the proteinuria will be divided into two classes for analysis: ≤0.5 g/24 h or >0.5 g/24 h. A logistic model with random effects will therefore be used [40]. The KTFS is a score that is computed at one year. The mean score at one year will be compared using a Student’s t-tests for paired data between the PPT and PDT groups.

The statistical significance level will be set at 5 % (type I error).

### Missing data considerations

The difficulties in dealing with missing data are often increased in the case of subjective data collected using questionnaires because missing data are often informative in this case. Indeed, we often observe that as health deteriorates, the risk of non-responses increases and missing data are therefore not random (missing not at random or MNAR). The analysis of MNAR data is truly problematic because it can lead to biased and inaccurate estimates of RS and of the true change of the subjective criteria one is trying to assess [42, 43]. Yet, methods such as SEM assume non informative missing data, that is to say missing at random (MAR) or completely missing at random (MCAR) data, unlike IRT models that can handle non-random missing data. The interest of IRT models was highlighted in cross-sectional and longitudinal studies [44–47]. These models were more powerful and allowed obtaining unbiased estimations of the effects of interest even in case of MNAR data, while models based on the observed scores showed poor properties in terms of bias and power estimates. The potential of using models coming from IRT, instead of or in addition to SEM models in the field of RS was also emphasized from a conceptual point of view [18, 48]. However, the formal assessment of the relative performance of SEM and IRT models in case of missing data has never been done to our knowledge. Hence, identification of the best methodological approach for RS detection in presence of missing data is still unknown. As stated in the Statistical analysis section, IRT and SEM will be used as complementary analysis and they will be also used to perform sensitivity analyses to assess the robustness of results.

## Ethical approval and registration

The study is registered with the ClinicalTrials.gov Registry (RC14_0078, NCT02154815), has approval from the ethical Committee for Persons’ Protection (CPP, Tours, 2014-S8), and from the advisory committee on research data and information in health (CCTIRS, Paris, 14.314).

## Discussion

The main assumption of our study is that preemptive kidney transplant recipients, who generally present a better level of health than patients undergoing dialysis, might although have a lower perceived quality of life and have more difficulties in adapting themselves to their new status. In contrast with the increasing number of studies in cancer, exploration of response shift has, to our knowledge, never been done in the context of ESRD despite its relevance in this disease, particularly in the context of preemptive transplantation. Our study will give more insight on the adjustment processes at hand in preemptive patients compared with patients with a quite short period under dialysis. Thanks to this knowledge, it might then be useful to organize a therapeutic educational program and psychological support specifically adapted for these preemptive patients before and following the transplant surgery. Exploration of the expectations of patients regarding transplantation could help personalize the specific aims of educational programs. It could increase the level of preparedness for transplantation and a better acceptance of its consequences. This point would be of major importance to prevent bad adherence to treatment if our results show that preemptive patients “regret” being transplanted because they are possibly not enough prepared to this new health status.

From a methodological point of view, the main limitation of our study may be the possible lack of comparability of the two groups that will be compared (PPT and PDT) despite matching on gender, center, and type of donor. To minimize this issue, all the statistical analyses, including all response shift analyses, will try to take into account confounding factors.

## Conclusion

The PreKit-QoL study is the first study which directly assesses the evolution of QoL and of other psychological criteria like anxiety or perceived stress of patients receiving a preemptive transplant taking patients’ adaptation into account through response shift analyses. We believe that this work may help providing a fresh perspective on expectations and care of transplanted patients, which may provide support for future research, care and preventive actions in the field of kidney transplantation. Our study might help to conceive specific and adapted individual and collective educational programs and psychological support in order to prevent a possible premature loss of the kidney as a consequence of non-compliance in patients that may be insufficiently prepared for transplantation.

## Abbreviations

QoL: Quality of Life
ESRD: End Stage Renal Disease
PPT: Patients with Preemptive Transplantation
PDT: Patients with Dialysis before Transplantation
PRO: Patient-Reported Outcomes
RS: Response Shift
GFR: Graft Filtration Rate
SEM: Structural equation models
IRT: Item Response Theory
MNAR: Missing Not At Random
MAR: Missing At Random
MCAR: Missing Completely At Random
